# Supplementary material for: Bioactive Glial-Derived Neurotrophic Factor from a Safe Injectable Collagen–Alginate Composite Gel Rescues Retinal Photoreceptors from Retinal Degeneration in Rabbits
Source: Mar Drugs. 2024 Aug 30;22(9):394. doi: 10.3390/md22090394 (PMC11433152; doi:10.3390/md22090394)
Supplement: Supplementary file 1 [file marinedrugs-22-00394-s001.zip › marinedrugs-3154104-supplementary.pdf]

Bioactive Glial-derived Neurotrophic Factor from A Safe Injectable Collagen–Alginate Composite Gel Rescues Retinal Photoreceptors from Retinal Degeneration in Rabbits

Tingyu Hu [1], Ting Zhou [1], Rajesh Kumar Goit [1,2], Ka Cheung Tam [1], Yau Kei Chan [1], Wai-Ching Lam [1,3] and Amy Cheuk Yin Lo [1,*]

1 Department of Ophthalmology, Li Ka Shing Faculty of Medicine, The University of Hong Kong, Hong Kong, China; tingyuhu@connect.hku.hk (T.H.); caroltz@connect.hku.hk (T.Z.); rajeshkgoit@gmail.com (R.K.G.); btkc0380@gmail.com (K.C.T.); jchanyk@hku.hk (Y.K.C.); waiching.lam@vch.ca (W.C.L.)
2 Jules Stein Eye Institute, Los Angeles, California, 90095, United States
3 Department of Ophthalmology and Visual Sciences, University of British Columbia, Vancouver, V5Z 3N9, Canada
* Correspondence: amylo@hku.hk; Tel.: (852) 28315363.

Category	Characterization Techniques
Safety	Electroretinography, Retinal Architecture Analysis, Retinal Stress Assessment, Biocompatibility (BIO) Evaluation
Physical Properties and Stability	Gel Morphology Analysis, Thickness Measurement, Internal Gel Structure Study
Biological and Drug Delivery Properties	Cell Viability and Proliferation Assays, Vitreous Glial Cell-Derived Neurotrophic Factor (GDNF) Analysis

Table S1: Summary of In Vivo Gel Characterization

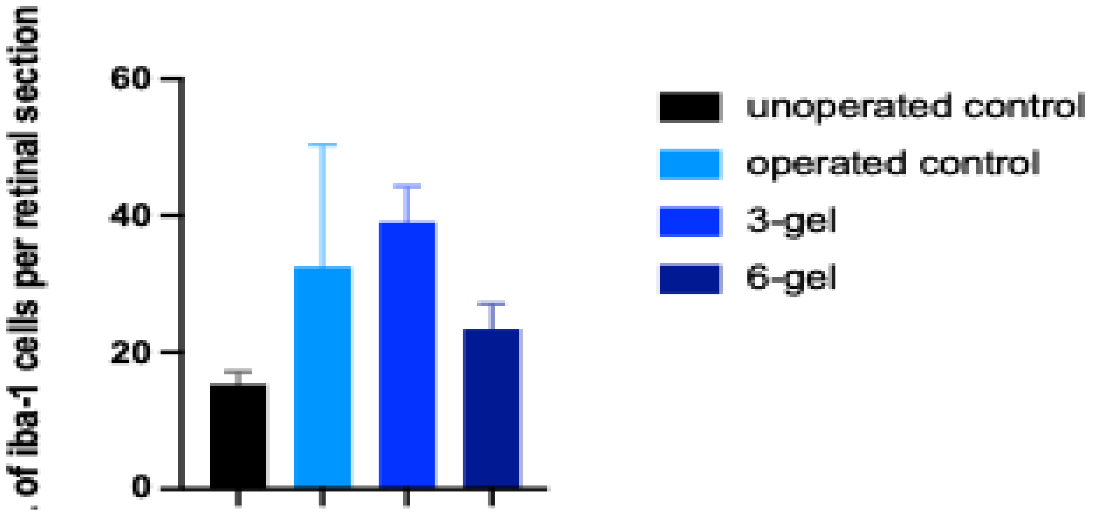

Figure S1: Quantitative analysis of IBA-1 positive cell counts per retina across different experimental groups. No significant differences were observed between the unoperated control, operated control, 3-Gel, and 6-Gel groups, as determined by One-way ANOVA followed by Bonferroni's post-hoc test (mean ± SEM; n = 4 for unoperated control, n = 3 for operated control, n = 5 for 3-Gel, and n = 5 for 6-Gel)

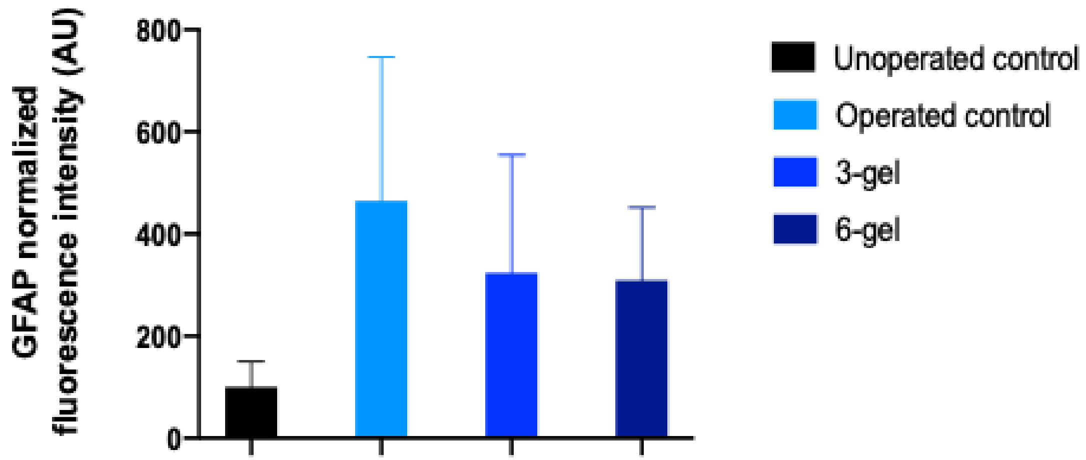

Figure S2: Immunohistochemical (IHC) fluorescence analysis of GFAP (Glial Fibrillary Acidic Protein) also revealed no significant changes among the unoperated control, operated control, 3-gel, and 6-gel groups. (mean ± SEM, n = 4, 3, 5, 5 for unoperated control, operated control, 3-gel and 6-gel respectively). One-way ANOVA followed by Bonferroni's post-hoc comparisons tests demonstrated no significant differences.

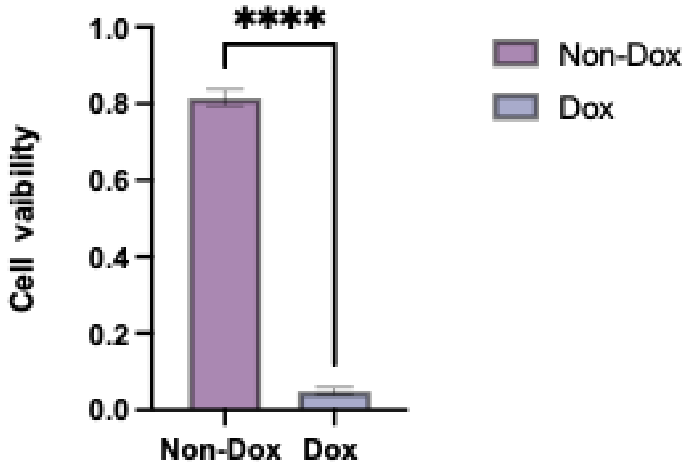

Figure S3: Viability of retrieved gels from Non-Dox and Dox group at 2 weeks assessed by Live-Dead assay (mean ± SD, n = 3, and 3 for Non-Dox and Dox respectively). Unpaired t-test demonstrated **** p <0.0001.

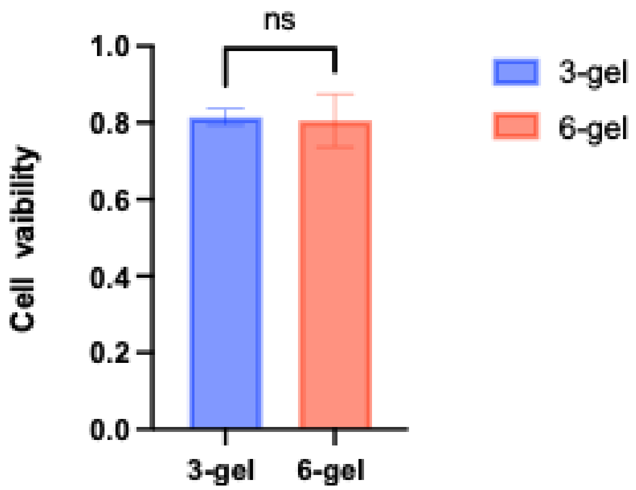

Figure S4: Viability of retrieved gels from 3-gel and 6-gel groups received with intravenous injection of SI at 2 weeks assessed by Live-Dead assay (mean ± SD, n = 3, and 3 for 3-gel and 6-gel respectively). Unpaired t-test demonstrated no significant differences, ns=not significant.
